# Apical Secretory Glycoprotein Complex Contributes to Cell Attachment and Entry by Cryptosporidium parvum

**DOI:** 10.1128/mbio.03064-22

**Published:** 2023-02-01

**Authors:** Marianna E. Akey, Rui Xu, Soumya Ravindran, Lisa Funkhouser-Jones, L. David Sibley

**Affiliations:** a Department of Molecular Microbiology, Washington University School of Medicine in St. Louis, St. Louis, Missouri, USA; Albert Einstein College of Medicine

**Keywords:** glycoprotein, lectin, mucin, adhesins, cell invasion, apicomplexan parasites

## Abstract

Cryptosporidium parvum is an enteric pathogen that invades epithelial cells in the intestine, where it resides at the apical surface in a unique epicellular location. Compared with those of related apicomplexan parasites, the processes of host cell attachment and invasion by C. parvum are poorly understood. The streamlined C. parvum genome contains numerous mucin-like glycoproteins, several of which have previously been shown to mediate cell attachment, although the majority are unstudied. Here, we identified the antigens recognized by monoclonal antibody (MAb) 1A5, which stains the apical end of sporozoites and mature merozoites. Immunoprecipitation with MAb 1A5 followed by mass spectrometry identified a heterodimer comprised of paralogous proteins which are related to additional orthologs in the genome of C. parvum and related species. Paralogous glycoproteins recognized by MAb 1A5 heterodimerize as a complex displayed on the parasite surface, and they also interact with lectins that suggest that they contain mucin-like, O-linked oligosaccharides. Although the gene encoding one of the paralogs was readily disrupted by CRISPR/Cas9 gene editing, its partner, which contains a mucin-like domain related to GP900, was refractory to deletion. Combined with the ability of MAb 1A5 to partially neutralize host cell attachment by sporozoites, these findings define a new family of secretory glycoproteins that participate in cell invasion by *Cryptosporidium* spp.

## INTRODUCTION

Cryptosporidium parvum is an enteric protozoan parasite that causes diarrheal disease in animals and zoonotic infections in humans (1). Cryptosporidiosis has its greatest impact on children in the developing world, where it is a major contributor to malnutrition and dehydration that can be fatal or lead to growth stunting (2, 3). Consistent with being a member of the phylum Apicomplexa, *Cryptosporidium* exhibits conserved apical features, such as the apical polar rings, as well as secretory organelles called micronemes (MIC), a single rhoptry (ROP), and dense granules (GRA) (4, 5). Despite the conservation of morphological features, few secretory proteins are recognized as orthologs based on BLAST homology, which may reflect the deep branching nature of this genus within the phylum (6) and/or rapid evolution of the constituents of secretory organelles. The few exceptions include C. parvum MIC1 (CpMIC1), a thrombospondin repeat-containing protein, and CpPRP1, a Sushi/SCR/CCP domain-containing protein (7), both identified by homology, and several ROP or rhoptry neck (RON) protein orthologs identified by cell fractionation and proteomics (8, 9).

Host cell invasion by C. parvum involves active motility driven by the parasite’s actin-myosin motor complex and is blocked when cytochalasin D is used to treat resistant host cells, implying a major role for parasite cytoskeleton in invasion (10). However, unlike most apicomplexans that inhabit vacuoles within the cytosol of the host cells, *Cryptosporidium* spp. are epicellular parasites, remaining enclosed within the host membrane but external to the cytosol (11). During and shortly after invasion, the host actin cytoskeleton is actively remodeled (12–14), a process likely aided by secretion of parasite ROP proteins such as ROP1, which was recently shown to interact with a LIM 7 only (LMO7) domain protein that controls epithelial host cell polarity (15).

MIC proteins in Toxoplasma gondii are known for their roles in cell adhesion; consistent with this role, they are enriched in domains that bind to a variety of host cell receptors, including oligosaccharide modifications like sialic acid and glycosaminoglycans (16). Similarly, prior studies on sporozoite invasion by C. parvum have emphasized the role of secretory glycoproteins such as GP900 (17, 18) and gp40/15 (19, 20) in cell attachment. Both antigens are recognized by monoclonal antibody (MAb) 4E9, which reacts with α-linked GalNac residues common to O-linked oligosaccharides (17). The MAb 4E9, as well as lectins that recognize α-GalNac residues, blocks C. parvum infection *in vitro*, demonstrating that carbohydrate interactions are important in host cell recognition (17). In addition to these two mucins, there are numerous mucin-like domain-containing proteins annotated in CryptoDB, suggesting that there is likely a large repertoire of adhesins in C. parvum. For example, mucins in a family known as CpMuc1 to CpMuc7 have been implicated in host cell attachment (21, 22). Additionally, many domains associated with adhesion in MIC proteins (i.e., mucin, thrombospondin [TSP], epidermal growth factor [EGF]-like, Apple, and Sushi/SCR/CCP domains) are also found in CryptoDB annotations. Characterization of C. parvum secretory proteins has lagged behind developments in other apicomplexans due to limitations in cultivation *in vitro* and availability of genetic tools. Recent developments in the use of CRISPR/Cas9 for gene editing (23) combined with methods for *in vitro* cultivation (24) have greatly accelerated progress in studying the basic biology of C. parvum.

A previous study that developed MAbs to intracellular stages of C. parvum identified several candidates that stain apical compartments in sporozoites and merozoites (25). Here, we characterized the antigens defined by MAb 1A5, which stains the apical end of sporozoites and mature merozoites (25). We demonstrate that 1A5 recognized a heterodimer of two related glycoproteins, one of which shares a mucin-like domain with GP900. The 1A5-reactive antigens were displayed on the surface of sporozoites, and MAb binding was able to partially block attachment, suggesting that the complex contributes to host cell recognition. Collectively, our study expands the repertoire of proteins involved in host cell attachment and invasion by C. parvum.

## RESULTS

### Identification of antigens apical glycoprotein 1 (AGP1) and AGP2 recognized by MAb 1A5.

**FIG 1:**
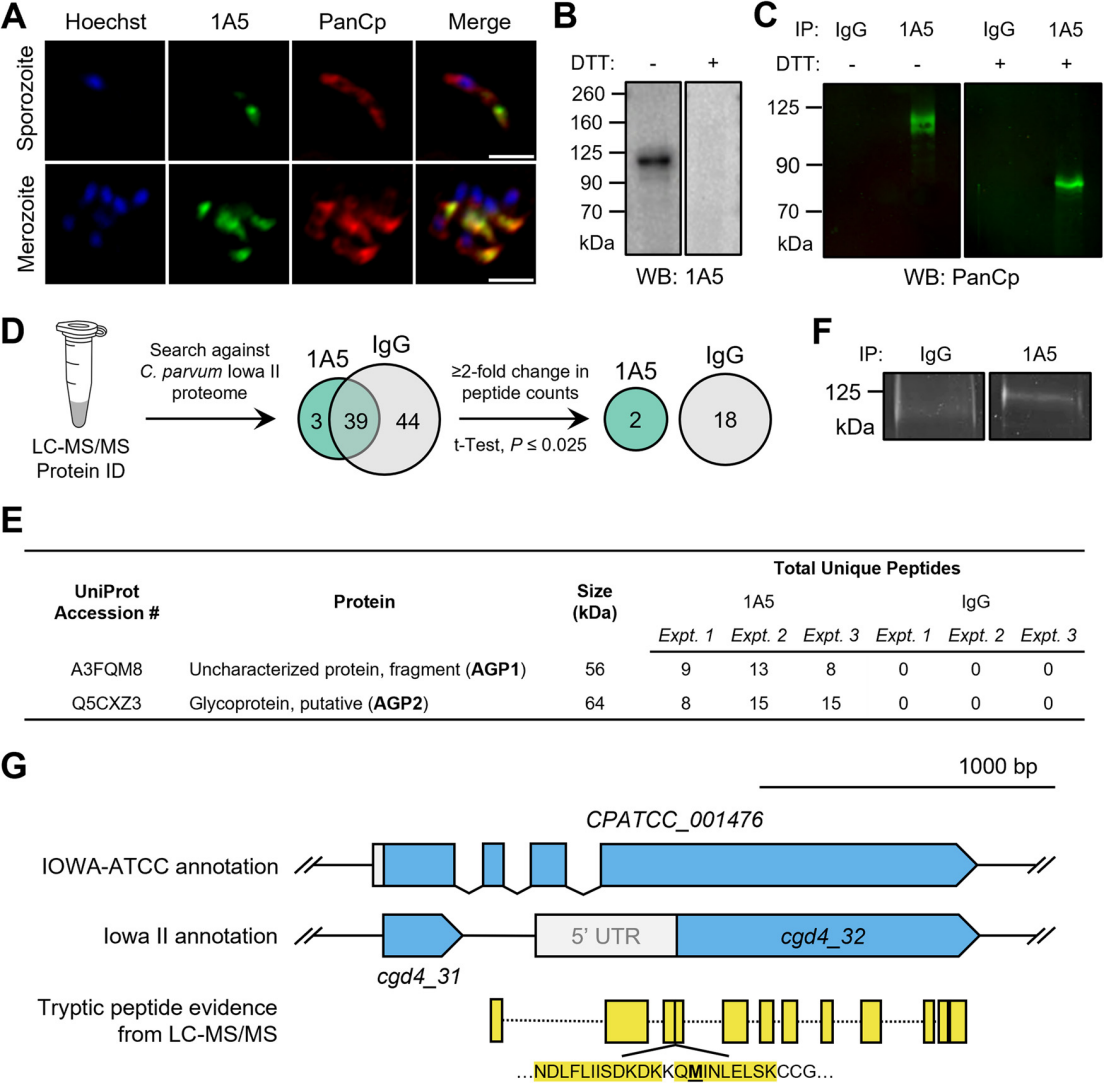
Identification of a glycoprotein complex recognized by MAb 1A5. (A) Immunofluorescence staining of sporozoites and merozoites with mouse MAb 1A5 followed by goat anti-mouse IgG–Alexa Fluor 488 and rabbit PanCp followed by anti-rabbit IgG–Alexa Fluor 568. Scale bars, 2 μm. (B) MAb 1A5 reactivity by Western blotting against sporozoite lysates in the presence or absence of the reducing agent DTT. MAb 1A5 detected a 125-kDa band in the absence of DTT. (C) Western blot detection using rabbit polyclonal PanCp of C. parvum proteins immunoprecipitated from sporozoite lysates by MAb 1A5 or mouse IgG control in the presence or absence of DTT. A 125-kDa antigen was observed in the nonreduced MAb 1A5 IP sample, and an 80-kDa band was observed in the reduced 1A5 IP sample. (D) Strategy for analysis of data from LC-MS/MS protein identification of IP samples. Identified proteins were first screened for C. parvum origin and then further filtered by a ≥2-fold change in total peptide counts in 1A5 versus IgG samples. Statistical significance was determined via Student’s *t* test analysis performed at a significance level of *P* of ≤ 0.05 with a Benjamini-Hochman correction for multiple tests. Adjusted significance level, *P* ≤ 0.025. C. parvum proteins identified by LC-MS/MS are found in Data Set S1 in the supplemental material. (E) Summary of C. parvum proteins identified as significantly enriched in 1A5 samples via LC-MS/MS analysis of three independent experiments. Predicted protein size was determined from the predicted mass of the sequence of the respective UniProt accession number. (F) SYPRO ruby staining of IgG and MAb 1A5 IP samples. The 125-kDa region was excised from both the IgG and 1A5 lanes for LC-MS/MS analysis. (G) Schematic of tryptic peptides (represented in yellow) from LC-MS/MS analysis aligned with the annotated ORFs of the predicted AGP1 locus (C. parvum Iowa II, *cgd4_32*; C. parvum IOWA-ATCC, *CPATCC_001476*). The peptide sequence that aligned with the 5′ end of the *cgd4_32* coding DNA sequence is shown, and the predicted start Met of the *cgd4_32* gene product is bolded and underlined. See Table S1 for tryptic peptide sequences.

To identify novel apical proteins in Cryptosporidium parvum, we sought to characterize the antigen of MAb 1A5, a mouse monoclonal antibody previously shown to stain mature merozoites (25). Initially, we evaluated the staining pattern by labeling parasites with 1A5 and PanCp, an anti-C. parvum polyclonal antiserum that labels all life stages of the parasite (24). Immunofluorescence staining with 1A5 labeled the apical compartment of both mature merozoites and sporozoites (Fig. 1A), which are both motile stages that invade host cells. Western blotting with 1A5 on sporozoite lysates revealed that MAb 1A5 recognized a 120-kDa protein in the absence of the reducing agent dithiothreitol (DTT) (Fig. 1B). The addition of DTT to the lysate sample ablated 1A5 binding, indicating that recognition of the epitope is dependent on an intact disulfide bond(s) (Fig. 1B).

10.1128/mbio.03064-22.4 TABLE S1 Tryptic peptides detected by LC-MS/MS for AGP1. Download Table S1, DOCX file, 0.02 MB. Copyright © 2023 Akey et al. 2023 Akey et al. https://creativecommons.org/licenses/by/4.0/ This content is distributed under the terms of the Creative Commons Attribution 4.0 International license.

10.1128/mbio.03064-22.6 DATA SET S1 Cryptosporidium parvum proteins detected by LC-MS/MS in IP samples. Download Data Set S1, XLSX file, 0.02 MB. Copyright © 2023 Akey et al. 2023 Akey et al. https://creativecommons.org/licenses/by/4.0/ This content is distributed under the terms of the Creative Commons Attribution 4.0 International license.

To identify the 120-kDa protein, we performed immunoprecipitation (IP) with MAb 1A5 and compared the results to those of mouse IgG as a negative control. Western blotting with rabbit PanCp detected the 120-kDa protein in the 1A5 IP sample resolved under nonreducing conditions (Fig. 1C). However, when the 1A5 IP sample was resolved under reducing conditions, PanCp labeled a previously unseen ~80-kDa protein (Fig. 1C). Samples from three independent IP experiments using IgG versus MAb 1A5 were then analyzed via liquid chromatography-tandem mass spectrometry (LC-MS/MS). Protein hits were filtered for C. parvum origin, followed by at least 2-fold enrichment and statistical significance determined using Student’s *t* test (Fig. 1D; see Data Set S1 in the supplemental material). Two proteins meeting these criteria were identified in the 1A5 IP samples: a 56-kDa uncharacterized protein fragment (UniProt number A3FQM8) and a 64-kDa putative glycoprotein (UniProt number Q5CXZ3) (Fig. 1E). Because neither of these proteins matched the expected size of the previously observed antigen, we sought to validate these hits by analyzing the protein composition of the 120-kDa band recognized by MAb 1A5 in nonreduced gels. Samples were immunoprecipitated using IgG versus 1A5 and resolved via nonreduced SDS-PAGE, and the resulting gel was stained with SYPRO ruby (Fig. 1F). The 120-kDa region from two independent gels was excised and examined via LC-MS/MS analyses. The same two C. parvum proteins were identified in the 120-kDa band isolated from the SYPRO ruby-stained gels (Data Set S2) and from the 1A5 IP described above.

10.1128/mbio.03064-22.7 DATA SET S2 Cryptosporidium parvum proteins detected by LC-MS/MS in gel slices. Download Data Set S2, XLSX file, 0.01 MB. Copyright © 2023 Akey et al. 2023 Akey et al. https://creativecommons.org/licenses/by/4.0/ This content is distributed under the terms of the Creative Commons Attribution 4.0 International license.

The proteins immunoprecipitated by MAb 1A5 were assigned the names apical glycoprotein 1 (APG1 [UniProt number A3FQM8]) and apical glycoprotein 2 (APG2 [UniProt number Q5CXZ3]) (Fig. 1E), based on their apical putative localization and previous characterization as glycoproteins (26). The UniProt accession numbers were cross-referenced with CryptoDB C. parvum Iowa II protein coding sequences, and proteins A3FQM8 and Q5CXZ3 were identified as the products of genes *cgd4_32* and *cgd7_4330*, respectively. However, we observed that the tryptic peptide sequences identified for AGP1 extended past the predicted start Met of the predicted protein encoded by *cgd4_32* (Fig. 1G). In fact, the first eight predicted residues of the *cgd4_32* gene product, MINLELSK, were found in a tryptic peptide that included a Gln residue preceding the predicted start Met (Fig. 1G; Table S1). After checking these sequences against alternative open reading frames (ORFs), we matched these unaligned peptides to *CPATCC_001476*, a predicted protein coding sequence from the C. parvum IOWA-ATCC genome annotation (27). *CPATCC_001476* encompasses ORFs from both *cgd4_31* and *cgd4_32*, along with a genomic region that was previously annotated as a noncoding intergenic region. As a result, the *CPATCC_001476* gene product is expected to be 66 kDa, which is closer in size to the protein fragment identified by LC-MS/MS. We favor the newer annotation *CPATCC_001476* as correct, as the prior description of *cgd4_32* does not match the expected size nor conform to the MS data. Collectively, our findings indicate that 1A5 IP recognizes a 120-kDa protein complex comprised of two antigens: AGP1, encoded by *CPATCC_001476*, and AGP2, encoded by *cgd7_4330*.

### AGP1 and AGP2 are paralogous members of a protein ortholog group unique to *Cryptosporidium* spp.

**FIG 2:**
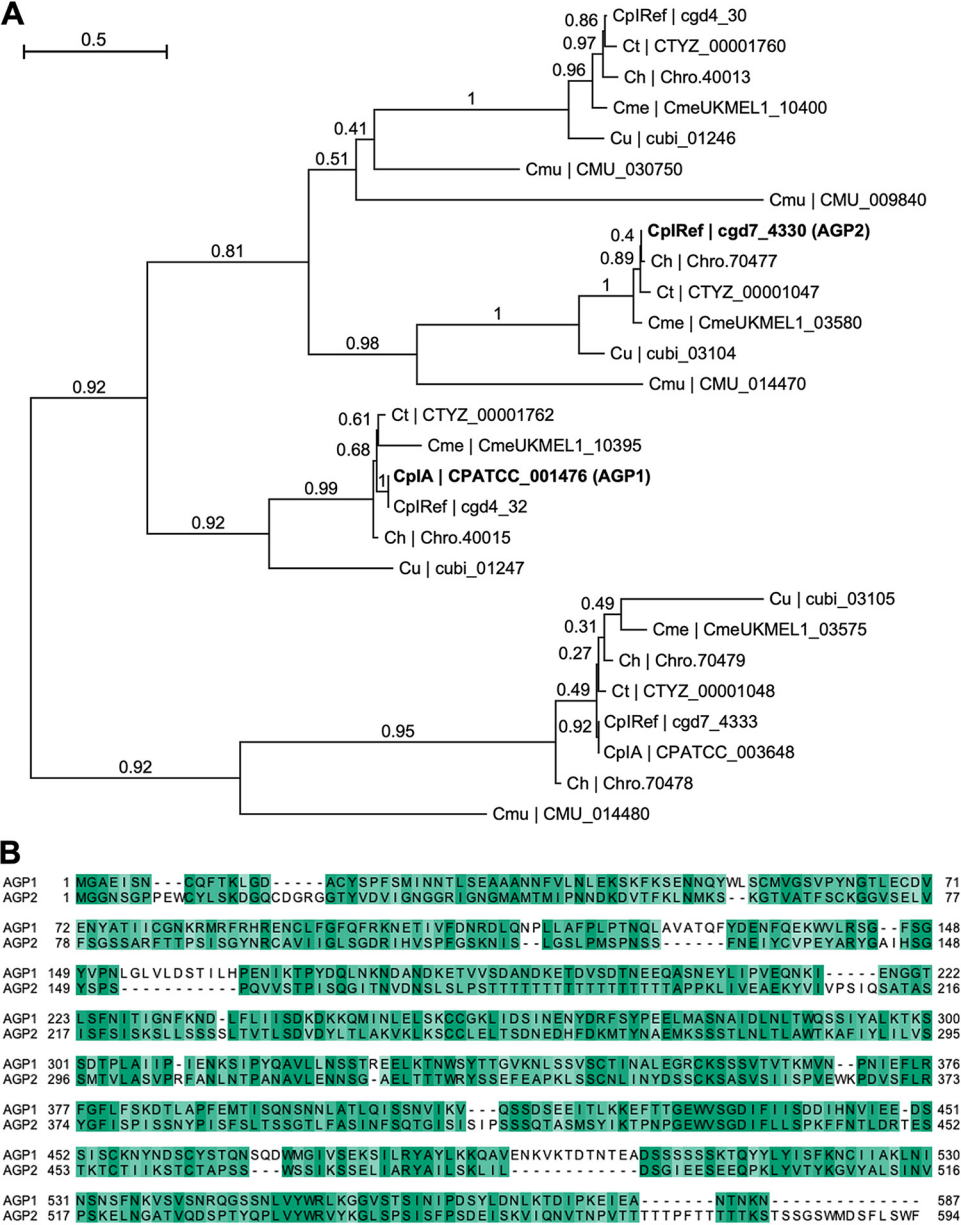
Phylogenetic analysis of AGPs. (A) Maximum likelihood phylogenetic tree of the paralogs AGP1 and AGP2 and their respective orthologs in five other *Cryptosporidium* species with branch support values. The *Cryptosporidium* species included are C. parvum Iowa (CpIRef, Iowa II annotation; CpIA, IOWA-ATCC annotation), C. hominis TU502 (Ch), *C. meleagridis* UKMEL1 (Cme), C. muris (Cmu), *C. tyzzeri* (Ct), and *C. ubiquitum* (Cu). CpIA protein sequence was included only if it differed from the CpIRef protein sequence. (B) Global pairwise alignment of protein sequences of the paralogs AGP1 and AGP2. Amino acid residues are colored by percent conservation.

The OrthoMCL database (28) grouped AGP1 and AGP2 into an ortholog group conserved among many *Cryptosporidium* species. This ortholog group included two additional hypothetical C. parvum proteins, encoded by *cgd4_30* and *cgd7_4333*. The four paralogs are encoded by two pairs of adjacent genes, one pair on chromosome 4 and one pair on chromosome 7. Phylogenetic analysis was performed on amino acid sequences from the four paralogous proteins in C. parvum and their respective homologs in the following *Cryptosporidium* species: C. hominis, C. meleagridis, C. muris, C. tyzzeri, and C. ubiquitum (Fig. 2A). Interestingly, the C. muris gene homologs of *AGP2* and *cgd7_4333* are syntenic with their C. parvum counterparts, a trait that is shared with only 4% of genes from major *Cryptosporidium* spp. (27). Conversely, no homologs in C. muris were identified for AGP1. We then directly compared the amino acid sequences of AGP1 and AGP2 with a global sequence alignment and found that the two sequences were determined to be 22.3% identical and 55.5% similar by using a BLOSUM50 scoring matrix (Fig. 2B).

### Validation of AGP1 and AGP2 as antigens recognized by 1A5.

**FIG 3:**
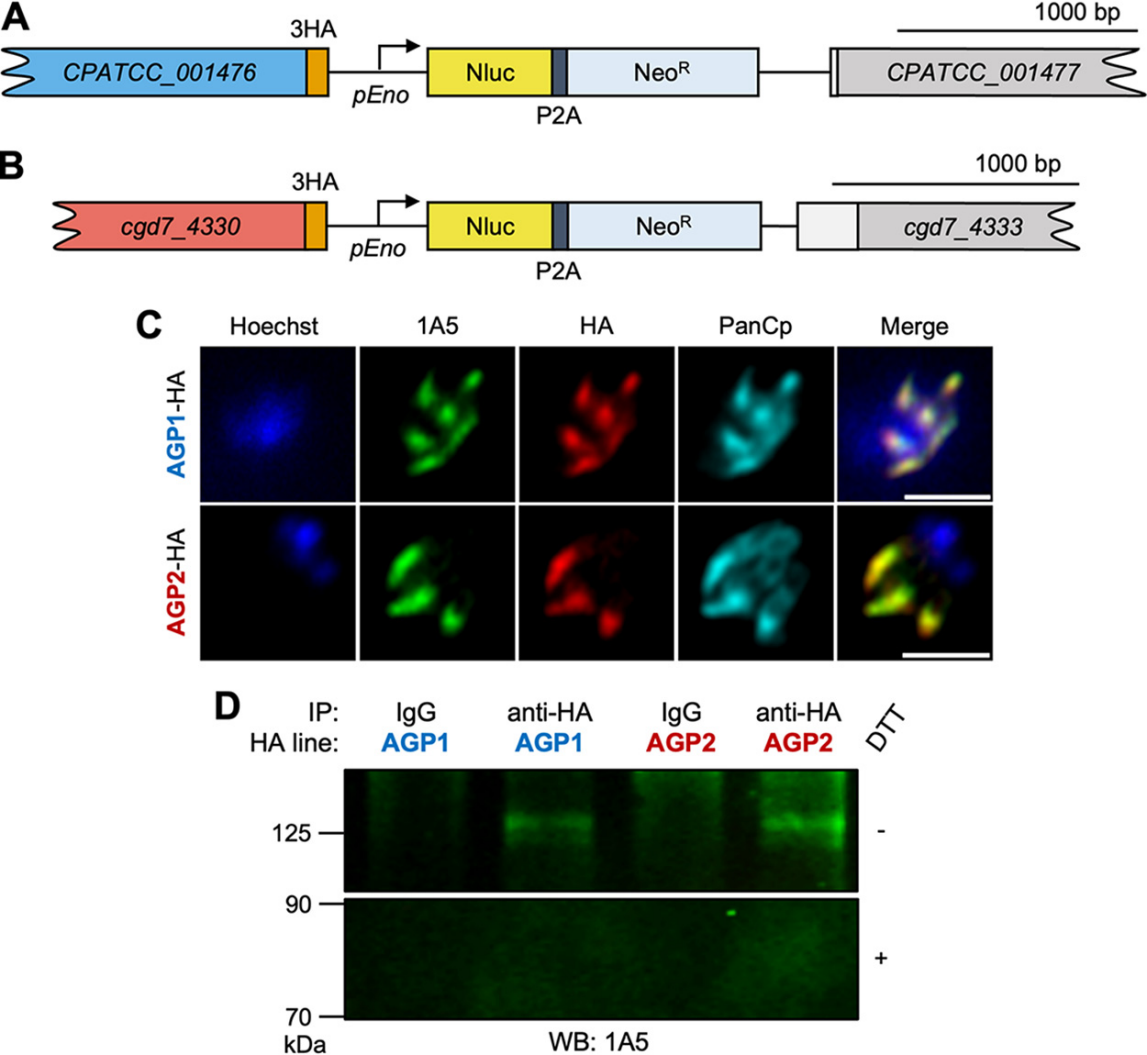
Validation of the glycoprotein complex recognized by 1A5. (A) Schematic of epitope-tagged AGP1-HA locus after CRISPR/Cas9 gene editing. (B) Schematic of epitope-tagged AGP2-HA locus after CRISPR/Cas9 gene editing. (C) Immunofluorescence staining of meronts from transgenic AGP1-HA and AGP2-HA lines stained with 1:500 MAb 1A5 followed by goat anti-mouse IgG–Alexa Fluor 488, 1:1,000 rabbit anti-HA followed by goat anti-rabbit IgG–Alexa Fluor 568, and 1:2,000 rat PanCp followed by goat anti-rat IgG–Alexa Fluor 647. The rat PanCp channel was not included in the merged images for clarity. HA labeling colocalized with MAb 1A5 labeling in both AGP1-HA and AGP2-HA meronts. Scale bars, 2 μm. (D) Western blot analysis of MAb 1A5 reactivity to proteins immunoprecipitated from AGP1-HA and AGP2-HA sporozoite lysates using rabbit anti-HA or rabbit IgG control. Samples were resolved by SDS-PAGE prior to Western blotting with MAb 1A5.

To confirm the successful capture of the antigen of MAb 1A5, we generated transgenic parasites expressing endogenously tagged AGP1-hemagglutinin (HA) (Fig. 3A) and AGP2-HA (Fig. 3B). We utilized CRISPR/Cas9 gene editing to add a triple hemagglutinin (3HA) tag and a Nluc-P2A-Neo^R^ selection cassette at the C terminus of either AGP1 or AGP2 (Fig. S1A and B), by following previously described strategies (29). Cas9/guide plasmids were designed for each construct with single guide RNA (sgRNA) sequences targeting the 3′ end of each respective ORF. We selected transgenic parasites by initial selection in *Ifng*^−/−^ (GKO) mice, followed by amplification in NOD *scid* gamma (NSG) mice treated with paromomycin (PRM) in drinking water, as described previously (29). Luciferase assays detected positive readings from fecal pellets at 6 days postinfection (dpi) (Fig. S1C), indicating the stable integration of repair templates. Oocyst shedding was quantified using quantitative PCR (qPCR), with peak shedding reached at 18 dpi (Fig. S1D). Proper integration of repair templates was confirmed via PCR analysis with amplicons specific to the template insertion regions of the AGP1- and AGP2-3HA-Nluc-P2A-Neo^R^ constructs (Fig. S1A, B, and E).

10.1128/mbio.03064-22.1 FIG S1 Strategy for the generation of AGP1-HA and AGP2-HA transgenic parasites. Schematics of strategy for tagging the AGP1 (A) and AGP2 (B) loci with 3HA and a Nluc-P2A-Neo^R^ resistance cassette. The constructs shown are as follows (from top to bottom): homology repair construct, native locus, and successfully modified locus. Locations of guide RNAs (AGP1 gRNA1 for AGP1-HA; AGP2 gRNA1 for AGP2-HA) and amplicons for PCR genotyping are also shown. Unannotated intergenic space used as part of the homology repair plasmid is represented by a thick line. (C) RLU per milligram of feces from nanoluciferase assays performed on fecal pellets collected from NSG mice infected with transgenic parasites. Each point represents a single sample from an individual mouse. Blue points represent samples taken from mice infected with AGP1-HA parasites (*n* = 6), and red points represent samples taken from mice infected with AGP2-HA parasites (*n* = 5). (D) Oocyst gDNA equivalents per milligram of feces from qPCR performed on DNA purified from fecal pellets collected from NSG mice infected with transgenic parasites. Each point represents a single sample from an individual mouse. Blue points represent samples taken from mice infected with AGP1-HA parasites (*n* = 6), and red points represent samples taken from mice infected with AGP2-HA parasites (*n* = 5). (E) PCR confirmation of correct integration of homology repair and resistance plasmid within the *AGP1* and *AGP2* loci. The AGP1 5′ Ins and AGP1 3′ Ins amplicons are specific for the 5′ and 3′ insertion sites, respectively, of the integrated AGP1-3HA-Nluc-P2A-Neo^R^-AGP1 construct. The AGP2 5′ Ins and AGP2 3′ Ins amplicons are specific for the 5′ and 3′ insertion sites, respectively, of the integrated AGP2-3HA-Nluc-P2A-Neo^R^-AGP2 construct. A short 318-bp sequence from an unrelated gene (*cgd3_3370*) was amplified as a positive control. Download FIG S1, TIF file, 0.7 MB. Copyright © 2023 Akey et al. 2023 Akey et al. https://creativecommons.org/licenses/by/4.0/ This content is distributed under the terms of the Creative Commons Attribution 4.0 International license.

To examine whether the tagged proteins colocalized with MAb 1A5, subcellular localization was examined in AGP1-HA and AGP2-HA parasites via immunofluorescence staining. Anti-HA stained the apical end of both AGP1-HA and AGP2-HA parasites and colocalized with MAb 1A5 staining (Fig. 3C). We then verified that AGP1 and AGP2 comprised the antigen recognized by 1A5 via IP followed by Western blot analysis. Sporozoite lysates were prepared from either AGP1-HA or AGP2-HA parasites and used for IP with rabbit IgG versus rabbit anti-HA antibody. IP samples were then Western blotted with 1A5, and the 120-kDa antigen was revealed in nonreduced anti-HA IP samples from both the AGP1-HA and AGP2-HA lysates (Fig. 3D). Similar to what was observed in Western blots with 1A5 on wild-type sporozoite lysates, no bands were observed in the reduced IP samples (Fig. 3D). These findings indicate that the 120-kDa 1A5 antigen contains both AGP1 and AGP2, which are held together by a disulfide bond that is necessary for recognition of the complex.

### Characterization of the AGP1-AGP2 heterodimer.

**FIG 4:**
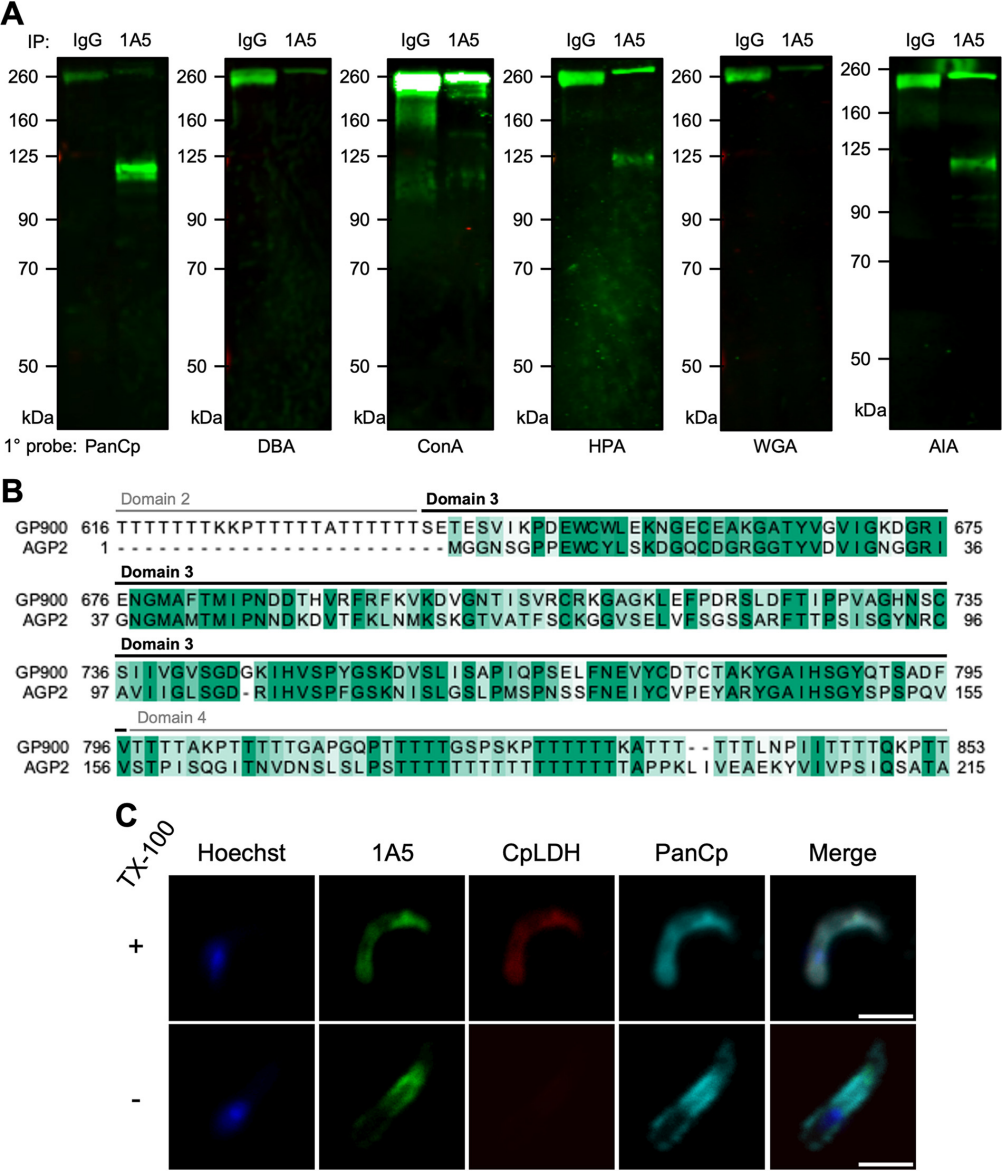
Functional characterization of the glycoprotein complex recognized by 1A5. (A) Lectin reactivity to samples immunoprecipitated (IP) using mouse IgG versus MAb 1A5 IP followed by separation by SDS-PAGE and transfer to nitrocellulose membranes. Blots were probed with PanCp or one of the following biotinylated lectins: Dolichos biflorus agglutinin (DBA), concanavalin A (ConA), *Helix pomatia* agglutinin (HPA), wheat germ agglutinin (WGA), or *Artocarpus integrifolia* agglutinin (AIA). Biotinylated lectins were detected using IRDye-conjugated streptavidin 800CW. (B) Local alignment of protein sequences of AGP2 and GP900. Amino acid residues are colored by percent conservation, and GP900 domains are annotated above each row of the alignment. (C) Immunofluorescence staining of permeabilized versus intact sporozoites with 1:250 1A5 followed by goat anti-mouse IgG–Alexa Fluor 488, 1:1,000 rabbit anti-CpLDH followed by goat anti-rabbit antibody, and 1:1,000 rat PanCp followed by goat anti-rat IgG–Alexa Fluor 647. Sporozoites were probed with primary and secondary antibodies in the presence (permeabilized) or absence (intact) of 0.05% TX-100 detergent. Anti-CpLDH was used as a cytosolic control to determine whether a sporozoite was intact or permeabilized. Scale bars, 2 μm.

After confirming that the AGP1-AGP2 heterodimer comprises the 1A5 antigen, we sought to further characterize the complex to determine its role in the parasite’s biology. Although both proteins lacked functional homology predictions, their apical localization, putative glycoprotein predictions, and specificity to mature invasive stages of the parasite implied that this complex may serve a function similar to that of other apical glycoproteins, which are known to mediate C. parvum attachment and invasion (17, 30). To investigate potential similarities in glycosylation patterns, IgG and 1A5 IP samples from wild-type sporozoite lysates were probed with rabbit PanCp or a panel of biotinylated lectins that label other characterized C. parvum glycoproteins (17): Dolichos biflorus agglutinin (DBA), concanavalin A (ConA), Helix pomatia agglutinin (HPA), wheat germ agglutinin (WGA), and Artocarpus integrifolia agglutinin (AIA). The AGP1-AGP2 heterodimer was selectively recognized by lectins reactive to α-GalNAc and α-Gal residues in T (Galβ1-3GalNAc) and Tn (GalNAc-α1-*O*-Ser/Thr) antigens: HPA and AIA (Fig. 4A). However, the protein complex was not reactive to DBA, another α-GalNAc-binding lectin specific to the Forssman antigen (GalNAcα1-3GalNAc), nor was it reactive to ConA and WGA lectins specific to d-glucose, GlcNAc, or mannose glycans (Fig. 4A). The aforementioned lectin-binding profile indicates that the AGP1-AGP2 heterodimer may contain O-linked glycosylation motifs, similar to other C. parvum secretory glycoproteins such as gp40 and the mucin-like GP900 (17).

To investigate potential sequence similarity of AGP1 and AGP2 to the previously studied glycoproteins GP900 and gp40/15, Smith-Waterman local pairwise alignments were created to identify regions of similarity between each pair while compensating for sequence length differences that would bias alignment scores. Although significant similarity was not observed in pairwise alignments containing either AGP1 or gp40/15, local pairwise alignment revealed high similarity between AGP2 and a 263-amino-acid region of GP900 (Fig. 4B; Fig. S2). Interestingly, the greatest proportion of overlap with AGP2 was observed in domain 3 of the mucin-like glycoprotein (Fig. 4B). In a previous study, recombinantly expressed domain 3 of GP900 competitively inhibited sporozoite invasion, indicating that this region participates in host cell attachment and invasion by GP900 (30).

10.1128/mbio.03064-22.2 FIG S2 Full local alignment of protein sequences of AGP2 and Gp900. Amino acid residues are colored by percent conservation, and Gp900 domains are annotated above each row of the alignment. Download FIG S2, TIF file, 1.7 MB. Copyright © 2023 Akey et al. 2023 Akey et al. https://creativecommons.org/licenses/by/4.0/ This content is distributed under the terms of the Creative Commons Attribution 4.0 International license.

Several invasion-related C. parvum MIC proteins associate with the sporozoite surface (19, 30). To investigate whether the same was true of the AGP1-AGP2 heterodimer, sporozoites were probed with 1A5, the cytosolic protein anti-*Cryptosporidium* lactose dehydrogenase (CpLDH) (31), and rat PanCp in the presence or absence of the detergent Triton X-100 (TX-100). Permeabilized sporozoites, stained after treatment with TX-100, displayed apical staining by 1A5, as previously observed (Fig. 4C). Intact sporozoites, stained in the absence of TX-100 and confirmed by lack of cytoplasmic CpLDH staining, displayed 1A5 staining at the surface of sporozoites, with a bias toward the apical end (Fig. 4C). Taken together, our findings indicate that the AGP1-AGP2 complex is localized to the surface of sporozoites and displays a lectin-binding profile consistent with O-linked glycosylation.

### Genetic disruption of AGPs.

**FIG 5:**
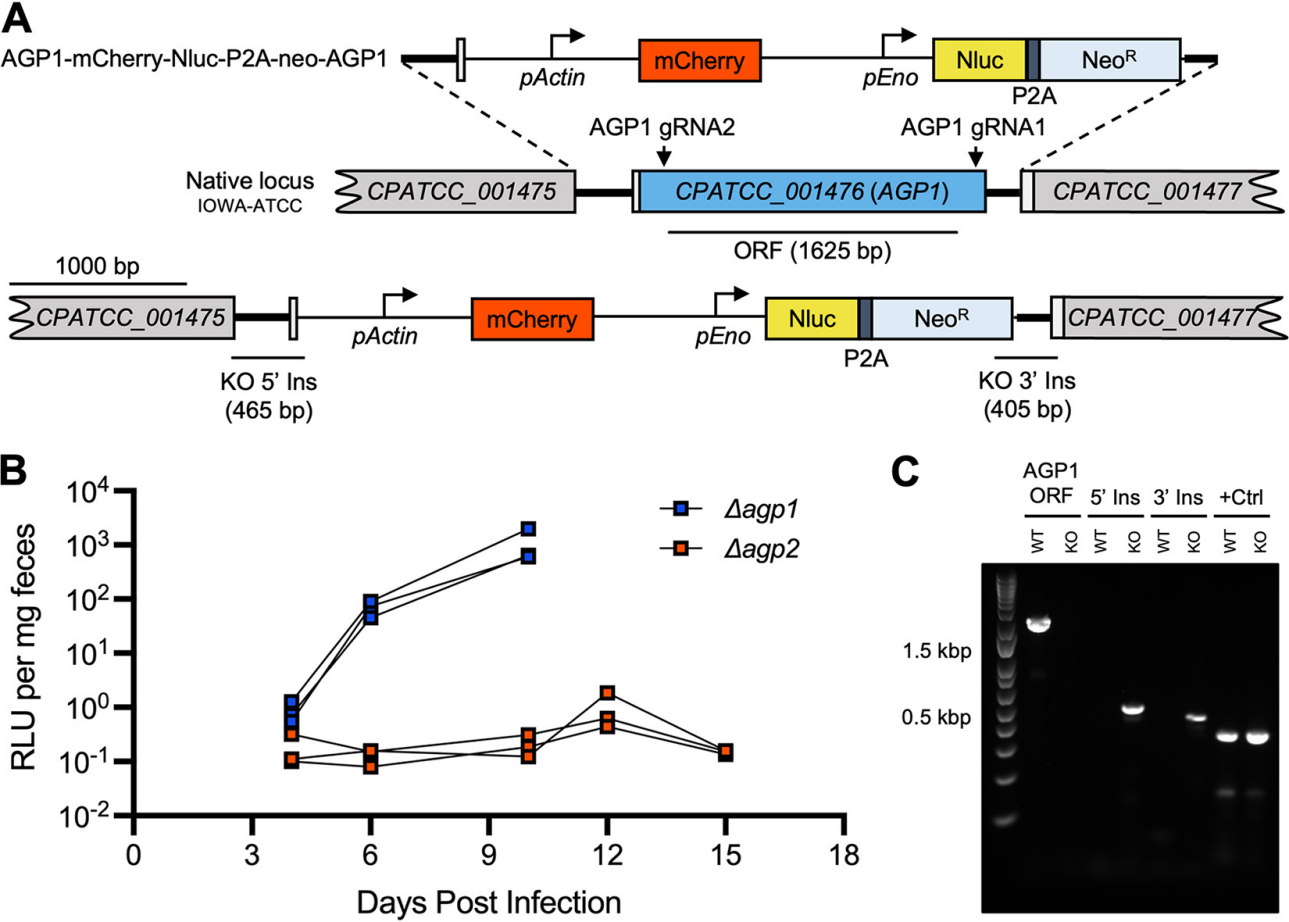
Genetic disruption of AGPs. (A) Schematics of strategy for genetic deletion of the *AGP1* locus. A construct was designed to replace the native locus with an mCherry reporter and an Nluc-P2A-Neo^R^ resistance cassette. The constructs shown are as follows (from top to bottom): homology repair construct, native locus, and successfully modified locus. Locations of dual guide RNAs (AGP1 gRNA1 and AGP1 gRNA2) and amplicons for Δ*agp1* PCR genotyping are also shown. Unannotated intergenic space used as part of the homology repair plasmid is represented by a thick line. (B) Relative luminescence units (RLU) per milligram of feces from nanoluciferase assays performed on fecal pellets collected from GKO mice infected with transfected sporozoites. Each point represents a single sample from an individual mouse. Blue points represent samples taken from mice infected with sporozoites cotransfected with the pAGP1-mCh-Nluc-P2A-NeoR-AGP1 repair and Cas9 plasmids (Δ*agp1*; *n* = 3). Red points represent samples taken from mice infected with sporozoites cotransfected with the pAGP2-mCh-Nluc-P2A-NeoR-AGP2 repair and Cas9 plasmids (Δ*agp2*; *n* = 3). All mice in the Δ*agp1* group were sacrificed by 11 days postinfection. (C) PCR confirmation of the Δ*agp1* knockout. The ORF amplicon is specific for a 1,625-bp fragment of the *CPATCC_001476* ORF. The 5′ Ins and 3′ Ins amplicons are specific for the 5′ and 3′ insertion sites, respectively, of the integrated AGP1-mCh-Nluc-P2A-Neo^R^-AGP1 construct. A short 318-bp sequence from an unrelated gene (*cgd3_3370*) was amplified as a positive control (+Ctrl).

To explore the role of the proteins recognized by 1A5, we attempted to generate transgenic parasite lines with genetic knockouts at either the *AGP1* (Δ*agp1*) (Fig. 5A) or *AGP2* (Δ*agp2*) (Fig. S3A) locus. CRISPR/Cas9 was used to replace the endogenous loci with an actin-promoter-driven mCherry protein and an Nluc-P2A-Neo^R^ selection cassette. Due to the predicted length of the *CPATCC_001476* ORF, the Cas9/guide plasmid for the Δ*agp1* construct contained two sgRNA sequences targeting either the 5′ or 3′ end of the ORF. The Cas9/guide plasmid for the construct was designed with the same sgRNA sequence used for the generation of the AGP2-HA construct. *Ifng^−/−^* mice (GKO) were infected in parallel with sporozoites cotransfected with plasmids targeting either the *agp1* or *agp2* loci, and fecal luciferase assays were performed to detect transgenic parasites. Positive luciferase readings were detected 6 dpi in mice infected with Δ*agp1* parasites, and all mice in the Δ*agp1* group were sacrificed by 10 dpi due to substantial weight loss due to parasite burden (Fig. 5B). Δ*agp1* parasites from GKO fecal samples were then amplified in NSG mice as described above. Fecal luciferase assays (Fig. S3B) and oocyst shedding (Fig. S3C) were quantified every 3 days. Interestingly, oocyst shedding in mice infected with Δ*agp1* parasites (Fig. S3C) was comparable to that of mice infected with AGP1-HA parasites (Fig. S1D). PCR analysis was used to confirm proper integration of the mCh-Nluc-P2A-Neo^R^ template and validate the deletion of the *agp1* ORF in Δ*agp1* parasites (Fig. 5C).

10.1128/mbio.03064-22.3 FIG S3 Strategy for the generation of Δ*agp1* and Δ*agp2* transgenic parasites. (A) Schematic of strategy for genetic deletion of the *AGP2* locus. A construct was designed to replace the native locus with an mCherry reporter and a Nluc-P2A-Neo^R^ resistance cassette. The constructs shown are as follows (from top to bottom): homology repair construct, native locus, and successfully modified locus. Locations of dual guide RNAs (AGP2 gRNA1 and AGP2 gRNA2) are also shown. Unannotated intergenic space used as part of the homology repair plasmid is represented by a thick line. (B) RLU per milligram of feces from nanoluciferase assays performed on fecal pellets collected from NSG mice (*n* = 3) infected with Δ*agp1* parasites. Each point represents a single sample from an individual mouse. (C) Oocyst gDNA equivalents per milligram of feces from qPCR performed on DNA purified from fecal pellets collected from NSG mice (*n* = 3) infected with Δ*agp1* parasites. Each point represents a single sample from an individual mouse. Download FIG S3, TIF file, 0.4 MB. Copyright © 2023 Akey et al. 2023 Akey et al. https://creativecommons.org/licenses/by/4.0/ This content is distributed under the terms of the Creative Commons Attribution 4.0 International license.

In contrast to the ease in which Δ*agp1* parasites were recovered, luciferase readings remained negative in all mice infected with Δ*agp2* parasites for the duration of the experiment (Fig. 5B). To increase transfection efficacy, a second Cas9 plasmid with dual sgRNA cassettes was created targeting two sites in the *AGP2* ORF (Fig. S3A). Two additional mouse infections with sporozoites transfected with the Δ*agp2* repair template and dual guide Cas9 plasmid were conducted, and again, luciferase readings were negative for the duration of each experiment. The repeated failures in the generation of Δ*agp2* parasites implied that *AGP2* may be refractory to gene deletion, despite the successful amplification of transgenic parasites endogenously tagged at the same locus (Fig. S1D).

### AGP1-AGP2 heterodimer contributes to host cell attachment and invasion.

**FIG 6:**
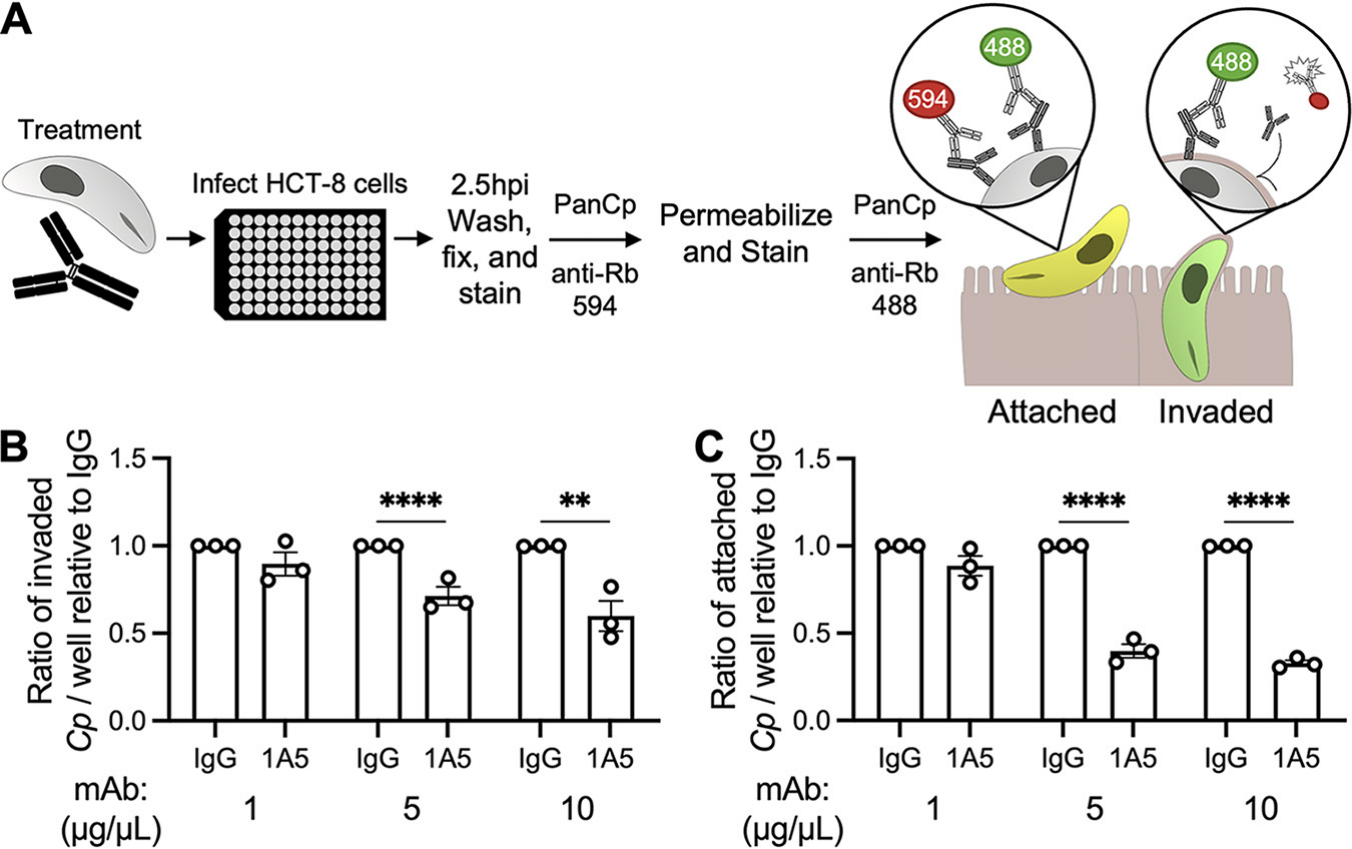
Role of the glycoprotein complex recognized by 1A5 in host cell attachment and invasion. (A) Schematic of workflow used to examine effects of antibody treatment on sporozoite attachment and invasion. HCT-8 cells were infected with treated sporozoites, fixed 2.5 hpi, and then immunofluorescence stained and imaged. Extracellular parasites (yellow) were labeled with both green and red fluorophores, while intracellular parasites (green) were labeled only with green fluorophores. (B and C) Average ratios of invaded (B) and attached (C) C. parvum parasites treated with 1A5 relative to those of mouse IgG controls at increasing antibody concentrations are shown. Data represent the combined mean ± standard error of the mean (SEM) of results of three independent experiments, with two to three technical replicates per experiment. Data were analyzed with a two-way ANOVA, followed by Dunnett’s test for multiple comparisons. **, *P *≤ 0.01; ****, *P *≤ 0.0001.

To determine whether the AGP1-AGP2 complex was involved in parasite invasion, we developed an assay to examine whether antibody binding inhibited either attachment or invasion by C. parvum sporozoites (Fig. 6A). Sporozoites were incubated with increasing concentrations of either mouse IgG or 1A5 and then allowed to infect HCT-8 cells for 2.5 h. After unattached sporozoites were removed by washing, samples were fixed and probed with rabbit PanCp, followed by goat anti-rabbit 594. Then, samples were permeabilized and again probed with rabbit PanCp, followed by goat anti-rabbit 488. Attached, but not invaded, parasites were labeled yellow, reflecting both steps in detection, while intracellular parasites were labeled only green. Intracellular and extracellular parasite numbers were quantified via automated imaging analysis, and 1A5-treated samples were normalized to the IgG control at each concentration. Both host cell invasion (Fig. 6B) and attachment (Fig. 6C) were significantly inhibited in a dose-dependent manner in samples treated with 1A5 compared to those treated with the IgG control. However, the inhibitory effect on sporozoite attachment was approximately 2-fold greater than the effect on invasion, indicating that 1A5 binding primarily obstructed parasite attachment and that the invasion defect was likely a downstream effect of impeded attachment.

## DISCUSSION

To define apical antigens of C. parvum, we characterized the antigen recognized by the previously described MAb 1A5, which stains the apical end of sporozoites and merozoites (25). Immunoprecipitation followed by mass spectrometry identified two related genes encoding the proteins AGP1 and AGP2, which comprise a heterodimer linked by a disulfide bond that collectively constitutes the 1A5 antigen. AGP1 and AGP2 are predicted glycoproteins that are part of a family of orthologs in *Cryptosporidium* spp., suggesting that they play a conserved role in this parasite. Lectin blots indicate that the AGP1-AGP2 complex is glycosylated and contains α-GalNAc and α-Gal residues, similar to other surface mucins in C. parvum (17–20). Genetic ablation studies revealed that AGP1 was dispensable, although AGP2 was refractory to disruption, suggesting that it plays an essential role. Consistent with the surface expression of AGP1-AGP2, MAb 1A5 inhibited sporozoite attachment to host cells, leading to decreased infection *in vitro*. Our findings identify a new glycoprotein complex that contributes to host cell invasion by C. parvum.

Sporozoites of C. parvum display substrate-dependent gliding motility (10, 32) similar to that of other apicomplexans (33). Parasite invasion of host cells is dependent on an actin-myosin motor in the parasite, as shown by the fact that cytochalasin D (CytD), which inhibits polymerization of actin, blocks invasion of C. parvum sporozoites into both wild-type and CytD-resistant host cells (10). Time-lapse video microscopy of the invasion process reveals that the membrane of the host cell envelopes the parasite, although the parasite remains at the apical pole of the host cell rather than entering the cytosol (10). During invasion, the parasite undergoes complex reorganization, which also evokes rapid changes in the actin cytoskeleton of the host (15), forming a pedestal of polymerized actin below the engulfed trophozoite (12–14, 34). Given the externally exposed surface localization on the apical end of sporozoites, it is likely that the AGP1-AGP2 complex acts at a very early step in this process to engage host receptors important for attachment.

The MAb 1A5 was generated in an earlier study to create a panel of stage-specific markers for the intracellular life stages of the parasite (25). Although other antibodies from the same study were reactive to the apical region of the parasite, 1A5 exclusively recognizes the apical compartment of mature merozoites and sporozoites, implying a specific temporal association of the 1A5 antigen with egress or invasion events. Previous studies have sought to identify invasion-related proteins via BLAST homology screens and functional domain predictions (7, 9, 35, 36). These methodologies for antigen discovery are complicated by the diversity of apical proteins across Apicomplexa, necessitated by various host and tissue tropisms. Instead, we sought to directly identify the antigen recognized by MAb 1A5 by purifying it from sporozoites. MAb 1A5 recognized an antigen of 120 kDa by Western blotting, and this reactivity was lost when the sample was reduced. Capture of proteins that were immunoprecipitated with 1A5 and analyzed by LC-MS/MS identified two novel apical proteins, AGP1 and AGP2, which comprise the antigen of 1A5. Surprisingly, each of these antigens was predicted to comprise proteins of only ~65 kDa, suggesting that the 120-kDa band may represent a heteromeric complex of the two individual proteins. The predicted heterodimer was independently validated by immunoprecipitating the complex, resolving it by SDS-PAGE, excising the 120-kDa band, and identifying the proteins by LC-MS/MS. Collectively, these findings indicate that the antigen recognized by MAb 1A5 comprises the proteins AGP1 and AGP2, which form a heterodimer mediated by a disulfide linkage, implicating a role for tertiary or quaternary structure in the epitope.

AGP1 and AGP2 are related to each other, and they also share similarity to a larger group of orthologs found in other *Cryptosporidium* spp. AGP2, which is located on chromosome 7 in C. parvum, has orthologs in a variety of related *Cryptosporidium* spp., including C. muris. Similarly, AGP1, which is located on chromosome 4, has other orthologs in most *Cryptosporidium* spp., except C. muris. In addition, there are two other closely related orthologous clusters, one on chromosome 4 (typified by *cgd4_30*) and one on chromosome 7 (typified by *cgd7_4333*). Collectively, these findings indicate that the various copies likely arose by duplication in a common ancestor, and they have slightly diverged since. Although IP and MS data indicate that AGP1 and AGP2 interact, the overall similarity of the proteins suggests that other pairwise complexes may also form. The normal growth of Δ*agp1* parasites *in vivo* suggests functional redundancy of this protein and predicts that its loss is unlikely to affect adherence and invasion *in vitro*. However, the inability to disrupt AGP2, despite the fact that we were able to tag the endogenous locus, suggests that AGP2 plays a more important role. Although we did not detect other partners in complex with AGP2, it may associate with other orthologs in the absence of AGP1 or perform an essential function on its own. Studies of essential genes in C. parvum are still challenging, although the function of AGP2 could be explored further in future studies using the recently developed conditional protein degradation genetic system (37).

AGP1 and AGP2 are both serine/threonine-rich proteins, similar to other mucins in C. parvum (17, 30). AGP1 is predicted to be a glycoprotein in addition to being serine/threonine rich (26). The hydroxyl residues of serine and threonine are typically used to attach O-linked oligosaccharides, and hence these residues are abundant in mucins. Consistent with this similarity, the AGP1-AGP2 complex reacted with lectins that recognize α-GalNAc and α-Gal residues in T (Galβ1-3GalNAc) and Tn (GalNAc-α1-*O*-Ser/Thr) antigens. AGP1-AGP2 also failed to stain with ConA and WGA, suggesting that it lacks N-linked oligosaccharides. Similar antigens have previously been defined in gp40 and GP900 (17, 30). Despite the similarity to other mucins in C. parvum, the epitope recognized by 1A5 is unlikely to recognize these core oligosaccharides, as shown by the fact that only AGP1 and AGP2 were immunoprecipitated and identified by MS analysis. Hence, the neutralizing ability of 1A5 is unlikely to be attributable to reaction to carbohydrates but rather is likely due to recognition of a unique conformational epitope in the complex. In contrast, previously characterized MAb 4E9, which directly recognizes α-GalNAc, reacts to gp40 and GP900, which are found on the surface of sporozoites, and this MAb neutralizes infectivity (17, 19, 20). GP900 has been localized to the micronemes using immuno-electron microscopy (30), and likely other mucins are also found in this compartment. The profile of 1A5 staining is also consistent with localization to micronemes, and studies using immunofluorescence analysis in the absence of detergent permeabilization revealed that it is also stably expressed on the sporozoite surface membrane. However, we were unable to confirm the localization at the electron microscopy (EM) level due to sensitivity of the epitopes to fixation. Nonetheless, micronemal localization is consistent with a function in adhesion, as shown from studies of other apicomplexans (16). Whether 1A5 directly disrupts a binding domain or merely causes steric hindrance that prevents interaction with a cognate receptor is uncertain from our studies. Nonetheless, the potent activity of 1A5 in blocking attachment suggests that the AGP1-AGP2 complex is involved in host recognition. Given its conservation across multiple *Cryptosporidium* spp., this function is likely to be conserved. Collectively, these studies expand the repertoire of apical secretory proteins that contribute to host cell invasion by C. parvum.

## MATERIALS AND METHODS

### Animal studies.

Studies on mice were approved by the Institutional Animal Studies Committee at the School of Medicine, Washington University in St. Louis. Commercially obtained *Ifng^−/−^* mice (GKO) (002287; Jackson Laboratories) and NOD *scid* gamma mice (NSG) (005557; Jackson Laboratories) were bred in-house at Washington University School of Medicine and were separated by sex after weaning. GKO mice, which are highly susceptible to the AUCP-1 strain of C. parvum used here, were used to isolate transgenic strains following the initial transfection of sporozoites. In contrast, NSG mice, which are more resistant, were used for prolonged infections to obtain sufficient oocysts for experiments. Mice were reared in a specific-pathogen-free facility on a 12-h-light:12-h-dark cycle with mouse chow and water *ad libitum*. For selection and amplification of transgenic C. parvum parasites, mice were given filtered tap water containing 16 g/L paromomycin sulfate salt (Biosynth International, Inc.). Animals that lost more than 20% of their body weight or became nonambulatory during infection were humanely euthanized.

### Cryptosporidium parvum parasites.

Cryptosporidium parvum AUCP-1 isolate oocysts were purified from fecal material after passage in Holstein calves, as previously described (38). Prior to use, purified oocysts were resuspended in a 40% (vol/vol) bleach solution (8.25% [wt/vol] sodium hypochlorite) and incubated for 10 min on ice. Oocysts were then washed three times in cold Dulbecco’s phosphate-buffered saline (DPBS). Bleached oocysts were resuspended in DPBS at 10^8^ oocysts/mL and stored at 4°C before use. For excystation, bleached oocysts were combined in a 1:1 solution with 1.5% (wt/vol) sodium taurocholate (Sigma) and incubated at 37°C for 1 h. At least 90% excystation was confirmed via microscopy before we proceeded with downstream experiments. To prepare lysates for Western blot analysis, sporozoites were resuspended in 2× Laemmli sample buffer and boiled at 95°C for 5 min. Lysates were centrifuged at 14,000 × *g* for 1 min at 4°C to remove insoluble material and then stored at −20°C before use.

### Generating rat PanCp polyclonal antiserum.

Antigen was generated by excysting 4 × 10^8^ oocysts as described above, and the cells were then freeze-thawed six times. The sample was then sent to Covance (Princeton, NJ, USA) for commercial antibody production. One rat was primed via subcutaneous injection with 200 μg antigen in Freund’s complete adjuvant, followed by boosting three times at 21-day intervals with 100 μg antigen and Freund’s incomplete adjuvant. The terminal bleed, referred to as rat PanCp antiserum, was used for indirect immunofluorescence experiments.

### HCT-8 cell culture and infection.

The human ileocecal colorectal adenocarcinoma cell line HCT-8 (ATCC CCL-244) was maintained in 10% fetal bovine serum (FBS) in RPMI 1640 medium modified to contain 10 mM HEPES, 1 mM sodium pyruvate, 4,500 mg/L glucose, and 1,500 mg/L sodium bicarbonate according to the ATCC formulation (ATCC-modified RPMI 1640; Thermo Fisher Scientific).

### Immunofluorescence microscopy.

For indirect immunofluorescence microscopy of extracellular sporozoites, 2 × 10^6^ sporozoites were added to coverslips coated with poly-l-lysine (Advanced Biomatrix) and centrifuged for 15 s at 400 × *g*. Adherent cells were fixed in 2% formaldehyde in DPBS at room temperature (RT) for 10 min and then blocked with 1% bovine serum albumin (BSA) in DPBS in the presence or absence of 0.05% Triton X-100 (TX-100). For immunofluorescence microscopy of intracellular stages of C. parvum, 2 × 10^6^ sporozoites were used to infect HCT-8 cells grown on coverslips. Cells were fixed 22 h postinfection (hpi) for 10 min at RT with 4% formaldehyde and then blocked and permeabilized with 1% BSA–0.1% TX-100 in PBS. Following fixation, cells were incubated with primary antibodies in 1% BSA in DPBS for 1 h at RT, washed with DPBS, and then incubated with secondary antibodies conjugated to Alexa Fluor dyes (Thermo Fisher Scientific) in 1% BSA in DPBS for 1 h at RT. Cells were washed with DPBS and then incubated with Hoechst nuclear dye (Thermo Fisher Scientific) diluted 1:2,000 in 1% BSA in DPBS for 20 min at RT. Coverslips were washed three times with DPBS and then mounted with ProLong glass antifade mountant (Thermo Fisher Scientific). Samples were viewed with a Zeiss LSM880 laser scanning confocal microscope (Carl Zeiss, Inc.) equipped with a 63×, 1.4-numerical-aperture Zeiss Plan Apochromat oil objective. Images were acquired using the ZEN 2.1 black edition software and edited in Fiji (https://imagej.net/software/fiji/).

### Immunoprecipitation.

Excysted sporozoites were lysed in 1% NP-40 lysis buffer (1% NP-40, 50 mM Tris HCl, 150 mM NaCl) for 30 min on ice with intermittent vortexing and then centrifuged at 14,000 × *g* for 15 min at 4°C to remove insoluble material. The supernatant was divided equally between samples and incubated with either mouse IgG (Sigma) or 1A5 on a tube rotator overnight at 4°C. Antibody-antigen solutions were added to protein G Dynabeads (Thermo Fisher Scientific) that had been washed with 0.02% Tween 20 (Thermo Fisher Scientific) in DPBS and then incubated on a tube rotator overnight at 4°C. Supernatants were removed from beads and stored at −20°C. Beads were washed with 0.02% Tween 20 in DPBS and then washed with DPBS. Immunoprecipitation (IP) bead samples were then stored at –80°C prior to LC-MS/MS processing or Western blot analysis.

### Western blot analysis.

Samples were boiled in 2× Laemmli sample buffer with or without 40 mM dithiothreitol (DTT; Sigma) at 95°C for 2 to 10 min, centrifuged at 5,000 × *g* for 30 s, resolved via SDS-PAGE, and transferred to nitrocellulose membranes. Membranes were blocked with Intercept blocking buffer (LI-COR) and then incubated with primary antibodies in 0.05% Tween 20 (Sigma) in PBS (PBST). Membranes were washed with PBST and then incubated with IRDye conjugated secondary antibodies (LI-COR) in PBST with 3% milk. Membranes were washed with PBST and imaged using an Odyssey imager (LI-COR) and Image Studio 3.0 software (LI-COR). Alternatively, membranes were incubated with primary antibodies, followed by HRP-conjugated goat anti-mouse antibody (Thermo Fisher Scientific) for 2 h at RT and washed with PBST. Membranes were incubated with Amersham ECL prime reagent (40:1 solution A-solution B; GE Healthcare) and then washed with PBST before imaging. Blots were viewed on the ChemiDoc MP imaging system (Bio-Rad) and imaged using Image Lab (Bio-Rad).

For lectin detection, samples were separated by SDS-PAGE and transferred to nitrocellulose membranes as described above. Membranes were blocked in PBS with 0.9 mM CaCl_2_ and 0.5 mM MgCl_2_ (PBS^++^) with 3% BSA overnight at 4°C. Membranes were then incubated with primary probes in 0.05% Tween 20 (Sigma) in PBS^++^ (PBST^++^). Membranes were washed with PBST^++^ and then incubated with IRDye conjugated secondary antibodies (LI-COR) in PBST^++^. Membranes were washed with PBST^++^ before imaging using an Odyssey imager (LI-COR) and Image Studio 3.0 software (LI-COR).

### SYPRO ruby staining and gel band excision.

Samples were immunoprecipitated using mouse IgG or MAb 1A5 and resolved on 10% SDS-PAGE gels. Gels were fixed two times for 30 min each in a 50% methanol–7% acetic acid solution and then incubated in SYPRO ruby gel stain (Thermo Fisher Scientific) overnight at RT. Gels were washed for 30 min in 10% methanol–7% acetic acid solution. Stained gels were viewed on a ChemiDoc MP imaging system (Bio-Rad) and imaged using Image Lab (Bio-Rad). Bands of interest were excised and stored at −80°C prior to LC-MS/MS processing.

### Identification of proteins via LC-MS/MS.

IP samples from three independent experiments and gel samples from two independent experiments were processed at the Nebraska Center for Biotechnology at University of Nebraska–Lincoln (Lincoln, NE, USA). Bead and gel samples were reduced at 37°C for 1 h in ammonium bicarbonate with 5 mM DTT and then alkylated for 20 min at 22°C in the dark using 15 mM iodoacetamine. The reaction was quenched with DTT and digested with trypsin overnight at 37°C. For gel samples only, peptides were extracted from gel fragments and redissolved in 2.5% acetonitrile–0.1% formic acid solution. Protein digests were analyzed with a Q-Exactive HF mass spectrometer (Thermo Fisher Scientific) using either a 1-h or a 2-h gradient on a 0.075-mm by 250-mm CSH C_18_ column (Waters). Samples were then searched against the following proteomes using Mascot 2.6.2 (v2.6.2, Matrix Science): Cryptosporidium parvum Iowa II (UniProt number UP000006726; 3,805 sequences), Bos taurus (UniProt number UP000009136; 37,512 sequences), Mus musculus (UniProt number UP000000589; 55,153 sequences), and the Global Proteome Machine common Repository of Adventitious Proteins (https://www.thegpm.org/). IP bead samples from the latter 2 independent replicates were also searched against the CryptoDB Cryptosporidium parvum IOWA-ATCC proteome (https://cryptodb.org, CryptoDB-48_CparvumIOWA-ATCC_AnnotatedProteins_20201109.fasta; 3,898 sequences). Scaffold (v4.8.9, Proteome Software, Inc.) was used to validate protein identifications. The validated data sets were then filtered by Cryptosporidium parvum origin using a protein threshold of 99.0%, a peptide threshold of 95%, and a minimum of 2 peptides. The fold change in peptide counts was determined using the ratio of total unique peptide counts in 1A5 samples relative to that in IgG samples averaged across two to three independent experiments. The average total unique peptide count was adjusted to 1 for proteins with no peptides detected in IgG samples for fold enrichment calculation. Statistical analyses were performed in Scaffold using Student’s *t* tests (*P < *0.05), followed by the Benjamini-Hochberg correction for multiple tests. C. parvum proteins identified by LC-MS/MS are listed in Data Set S1 and Data Set S2 in the supplemental material.

### Sequence alignment and analysis.

Amino acid sequences were obtained from CryptoDB (https://cryptodb.org). Global and pairwise alignments were created using the GGSEARCH2SEQ and SSEARCH2SEQ tools, respectively, using the BLOSUM50 matrix, and alignment graphics were created using Jalview (v2.11.2.2; https://www.Jalview.org) (39). Selected proteins from OG6_158731 (OrthoMCL database) were aligned using MUSCLE. Phylogenetic trees were constructed from this alignment using PhyML 3.1/3.0 aLRT (https://www.Phylogeny.fr) (40) with 100 replications for bootstrap analysis, and tree graphics were created and edited using TreeGraph2 (41).

### Construction of repair templates and CRISPR/Cas9 plasmids for genetic modification of C. parvum.

Repair templates for both 3HA tagging and gene knockouts were constructed using Gibson assembly with fragments amplified from previously described plasmids (24, 29) and homology regions amplified from purified C. parvum AUCP-1 genomic DNA (gDNA). CRISPR/Cas9 plasmids were generated via Q5 site-directed mutagenesis (New England BioLabs, Inc.) by replacing the thymidine kinase (TK) single-guide RNA (sgRNA) of pACT1:Cas9-GFP, U6:sgTK (Addgene plasmid no. 122852) (24) with a sgRNA targeting the gene of interest. To generate CRISPR/Cas9 plasmids for gene knockouts, a second U6:tracrRNA motif containing sgRNA1 was inserted into pACT1:Cas9-FLAG, U6:sgRNA2 via Gibson assembly to create pACT1:Cas9-FLAG, dual U6:sgRNA-KO. Plasmids and primers were designed using SnapGene software (v6.0.5; Insightful Science). sgRNAs were designed using the Eukaryotic Pathogen CRISPR guide RNA/DNA Design tool (EuPaGDT; http://grna.ctegd.uga.edu). Plasmids and primers are listed in Table S2.

10.1128/mbio.03064-22.5 TABLE S2 Key resources and oligonucleotides used in this study. Download Table S2, XLSX file, 0.02 MB. Copyright © 2023 Akey et al. 2023 Akey et al. https://creativecommons.org/licenses/by/4.0/ This content is distributed under the terms of the Creative Commons Attribution 4.0 International license.

### Generation and amplification of transgenic C. parvum parasites.

Excysted sporozoites were cotransfected with 50 μg repair template and 30 μg CRISPR/Cas9 targeting plasmid, as previously described (24, 29). Electroporated sporozoites were transferred to cold DPBS and stored on ice until infection. To select for transgenic parasites, three GKO mice per transgenic parasite line were administered 8% (wt/vol) sodium bicarbonate by oral gavage for 5 min prior to infection and then subsequently administered 2.5 × 10^7^ electroporated sporozoites in DPBS by oral gavage. Paromomycin (16 g/L; Biosynth International, Inc.) was added to drinking water 24 hpi. Fecal pellets were collected at 3-day intervals and stored at −80°C for DNA extraction or at 4°C for luciferase assays or for subsequent infections. Following confirmation of proper template integration, a fecal slurry containing 2 × 10^4^ oocysts from an infected GKO mouse was used to infect three to six NSG mice, as described previously (29). Paromomycin (16 g/L; Biosynth International, Inc.) drinking water was offered continuously beginning immediately after infection, and fecal pellets were isolated and tested as described above.

### Luciferase assay.

Luciferase assays were performed on fecal samples using the Nano-Glo luciferase assay kit (Promega) as previously described (29). Glass bead-extracted samples were centrifuged at 16,000 × *g* for 1 min and analyzed for luciferase activity using a Cytation 3 cell imaging multimode reader (BioTek). The number of relative luminescence units per milligram of feces was calculated using the average luminescence reading of two technical replicates per sample divided by the mass of the fecal sample used for the luciferase assay.

### Quantification of C. parvum oocyst shedding.

DNA was extracted from fecal samples using the QiaAmp PowerFecal pro DNA kit (Qiagen). qPCR was performed with the QuantStudio 3 system (Thermo Fisher Scientific) using previously described cycling conditions (24). Each sample was run as two technical replicates containing 2 μL purified fecal DNA (diluted 1:5 in low-Tris-EDTA buffer), 10 μL SYBR green QuickStart Taq ReadyMix (Sigma), and 1.6 μL of 5 μM primer solution targeting C. parvum GAPDH (glyceraldehyde-3-phosphate dehydrogenase) (Table S2). C. parvum oocyst quantities were determined via the QuantStudio Design & Analysis New (DA2) software (Thermo Fisher Scientific) using standard curves for C. parvum gDNA. The number of oocysts per milligram of feces was calculated as the average number of C. parvum oocyst gDNA equivalents (total gDNA equivalents divided by 4) divided by the mass of the fecal sample from which DNA was purified.

### PCR genotyping of transgenic oocysts.

To confirm proper template integration after CRISPR/Cas9-mediated genetic modification, PCR was used to amplify genomic regions of transgenic or wild-type parasites. PCR was performed using 0.8 μL purified fecal DNA template using Q5 hot start high-fidelity 2× master mix (New England BioLabs, Inc.) and primers at a final concentration of 500 nM. Reactions were performed with the following cycling conditions: 98°C for 30 s; 35 cycles of 98°C for 15 s, primer-specific annealing temperature for 30 s, and 72°C for 30 s; and a final extension of 72°C for 2 min. For the APG1 ORF amplicon, PCR was performed on 1 μL purified fecal DNA template using PrimeSTAR GXL premix (TaKaRa) and primers at a final concentration of 250 nM. Reactions were performed with the following cycling conditions: 98°C for 30 s; 35 cycles of 98°C for 10 s, 60°C for 15 s, and 68°C for 1.6 min; and a final extension of 72°C for 2 min. All PCRs were performed using the Veriti 96-well thermal cycler (Applied Biosciences). PCR products were resolved on TBE-agarose gels with 1:10,000 GelRed (Biotium). Gels were viewed on the ChemiDoc MP imaging system (Bio-Rad) and imaged using Image Lab (Bio-Rad). Primers are listed in Table S2.

### Invasion-attachment assay with antibody treatment.

Excysted sporozoites (2 × 10^5^) were resuspended in 4% FBS–ATCC-modified RPMI 1640 medium. Purified 1A5 and mouse IgG control antibodies were serially diluted in RPMI 1640 medium and added to a separate 96-well plate, and sporozoite solution was added to diluted antibodies at a 1:1 ratio. Sporozoite-antibody solutions were incubated at RT for 10 min and then added to confluent HCT-8 cells in a 96-well plate. Plates were centrifuged at 400 × *g* for 15 s and then incubated for 2.5 h. At 2.5 hpi, all wells were washed twice with DPBS, fixed with 2% formaldehyde in DPBS, washed with DPBS, and blocked with 1% BSA in DPBS. Extracellular C. parvum parasites were labeled with rabbit PanCp, followed by goat anti-rabbit IgG–Alexa Fluor 594. Wells were then permeabilized with 1% BSA–0.05% TX-100 in DPBS. After permeabilization, all C. parvum parasites were labeled with rabbit PanCp, followed by goat anti-rabbit IgG–Alexa Fluor 488. Nuclei were stained with Hoechst 33342 nuclear dye (Thermo Fisher).

Plates were imaged on the Cytation 3 cell imaging multimode reader (BioTek) using a 10× objective. Nine images were obtained per well using a 3-by-3 grid. Gen5 software (v5.08; Biotek) was used to count host cell nuclei in the DAPI (4′,6-diamidino-2-phenylindole) channel. To assess C. parvum parasite attachment and invasion, CellProfiler (v4.2.1; https://CellProfiler.org) (42) was used to count all objects in the green fluorescent protein (GFP) channel, and then the intensity of each object in the Texas Red channel was measured. Parasites were determined to be extracellular if the object from the GFP channel surpassed a fluorescence intensity threshold in the Texas Red channel. The number of invaded parasites was calculated by subtracting the number of extracellular parasites from the total number of parasites. Relative parasite invasion and attachment were calculated as a ratio of the mean number of invaded or extracellular C. parvum parasites, respectively, in 1A5 to that in the mIgG control. Sample means were averaged across three independent experiments with two to three technical replicates per experiment. Prism 9 (GraphPad) was used to perform a two-way analysis of variance (ANOVA), followed by Dunnett’s test for multiple comparisons, in which 1A5 was compared to mIgG within each concentration tested.

### Statistics.

Statistical comparisons were made using Prism 9 (GraphPad) to analyze whether the data were normally distributed, and appropriate nonparametric or parametric tests were chosen. Details of the specific test and significance values are given in the figure legends.

### Data availability.

All of the data associated with this study are found in this article or the supplemental material.
