# Supplementary material for: Apical Secretory Glycoprotein Complex Contributes to Cell Attachment and Entry by Cryptosporidium parvum
Source: mBio. 2023 Feb 1;14(1):e03064-22. doi: 10.1128/mbio.03064-22 (PMC9973360; doi:10.1128/mbio.03064-22)
Supplement: TABLE S1 [file mbio.03064-22-s0001.docx]

**Table S1 Tryptic peptides detected by LC-MS/MS for AGP1**

| # | **Unique Tryptic Peptides** | **Predicted CDS** | | **# Peptides Observed** | | | **Protein ID Probability** |
| --- | --- | --- | --- | --- | --- | --- | --- |
|  |  | ***cgd4_32*** | ***CPATCC_001476*** | **Expt. 1** | **Expt. 2** | **Expt. 3** |  |
| 1 | HRENCLFGFQFR*^a^* | N | Y | 0 | 1 | 0 | 100% |
| 2 | ENCLFGFQFR*^a^* | N | Y | 0 | 1 | 1 | 100% |
| 3 | TPYDQLNK | N | Y | 1 | 0 | 1 | 100% |
| 4 | TPYDQLNKNDANDKETVVSDANDKETDVSDTNEEQASNEYLIPVEQNK | N | Y | 0 | 1 | 0 | 100% |
| 5 | NDANDKETVVSDANDKETDVSDTNEEQASNEYLIPVEQNK | N | Y | 0 | 1 | 1 | 100% |
| 6 | NDLFLIISDKDK | N | Y | 0 | 1 | 1 | 100% |
| 7 | QMINLELSK | N | Y | 1 | 1 | 1 | 100% |
| 8 | SSDTPLAIIPIENK | Y | Y | 1 | 1 | 0 | 100% |
| 9 | SIPYQAVLLNSSTR | Y | Y | 1 | 1 | 1 | 100% |
| 10 | NLSSVSCTINALEGR | Y | Y | 1 | 1 | 1 | 100% |
| 11 | MVNPNIEFLR | Y | Y | 0 | 1 | 0 | 100% |
| 12 | VQSSDSEEITLK | Y | Y | 0 | 1 | 0 | 100% |
| 13 | VQSSDSEEITLKK | Y | Y | 2 | 2 | 2 | 100% |
| 14 | NYNDSCYSTQNSQDWMGIVSEK | Y | Y | 2 | 2 | 2 | 100% |
| 15 | QGSSNLVYWR | Y | Y | 1 | 1 | 0 | 100% |
| 16 | GGVSTSINIPDSYLDNLK | Y | Y | 1 | 1 | 0 | 100% |

*^a^*Peptides 1 and 2 are present only in the complete *CPATCC_001476* gene product predicted by the *C. parvum* IOWA-ATCC proteome and absent from the A3FQM8_CRYPI protein fragment predicted by the Uniprot *C. parvum* Iowa II proteome.
