# Supplementary figures and images for: Apical Secretory Glycoprotein Complex Contributes to Cell Attachment and Entry by Cryptosporidium parvum
Source: mBio. 2023 Feb 1;14(1):e03064-22. doi: 10.1128/mbio.03064-22 (PMC9973360; doi:10.1128/mbio.03064-22)

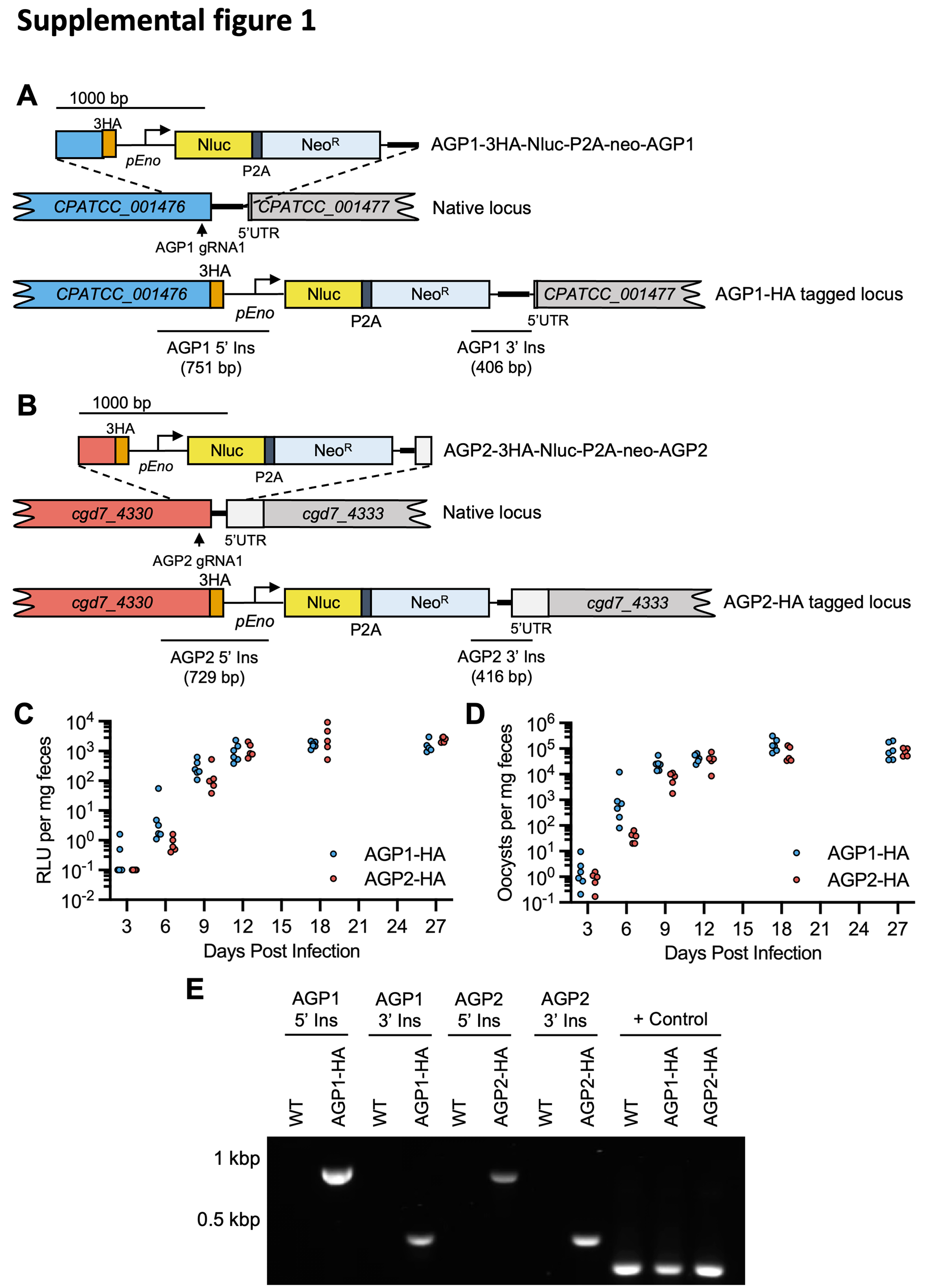

Supplement: FIG S1 [file mbio.03064-22-s0005.tif]

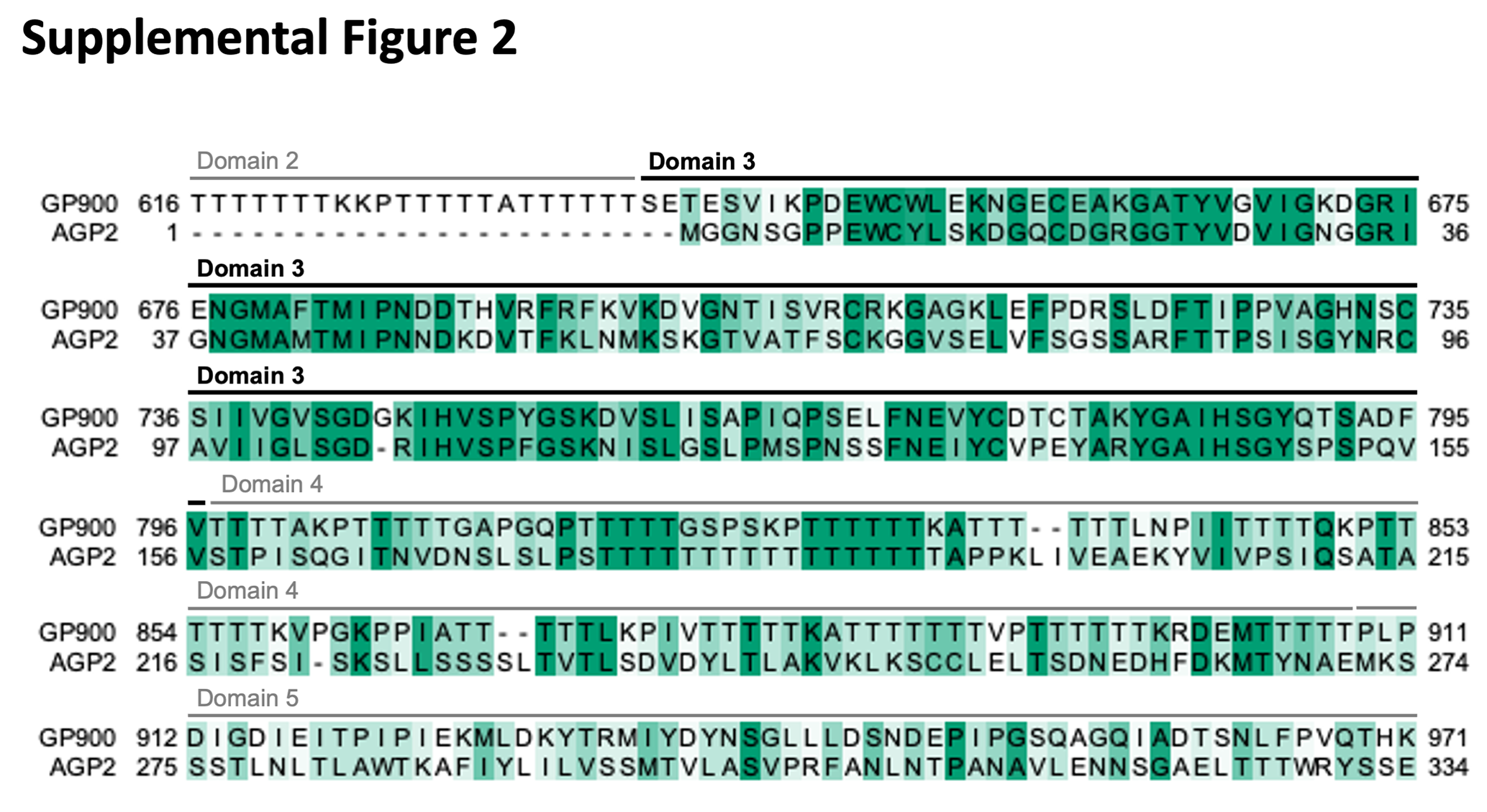

Supplement: FIG S2 [file mbio.03064-22-s0006.tif]

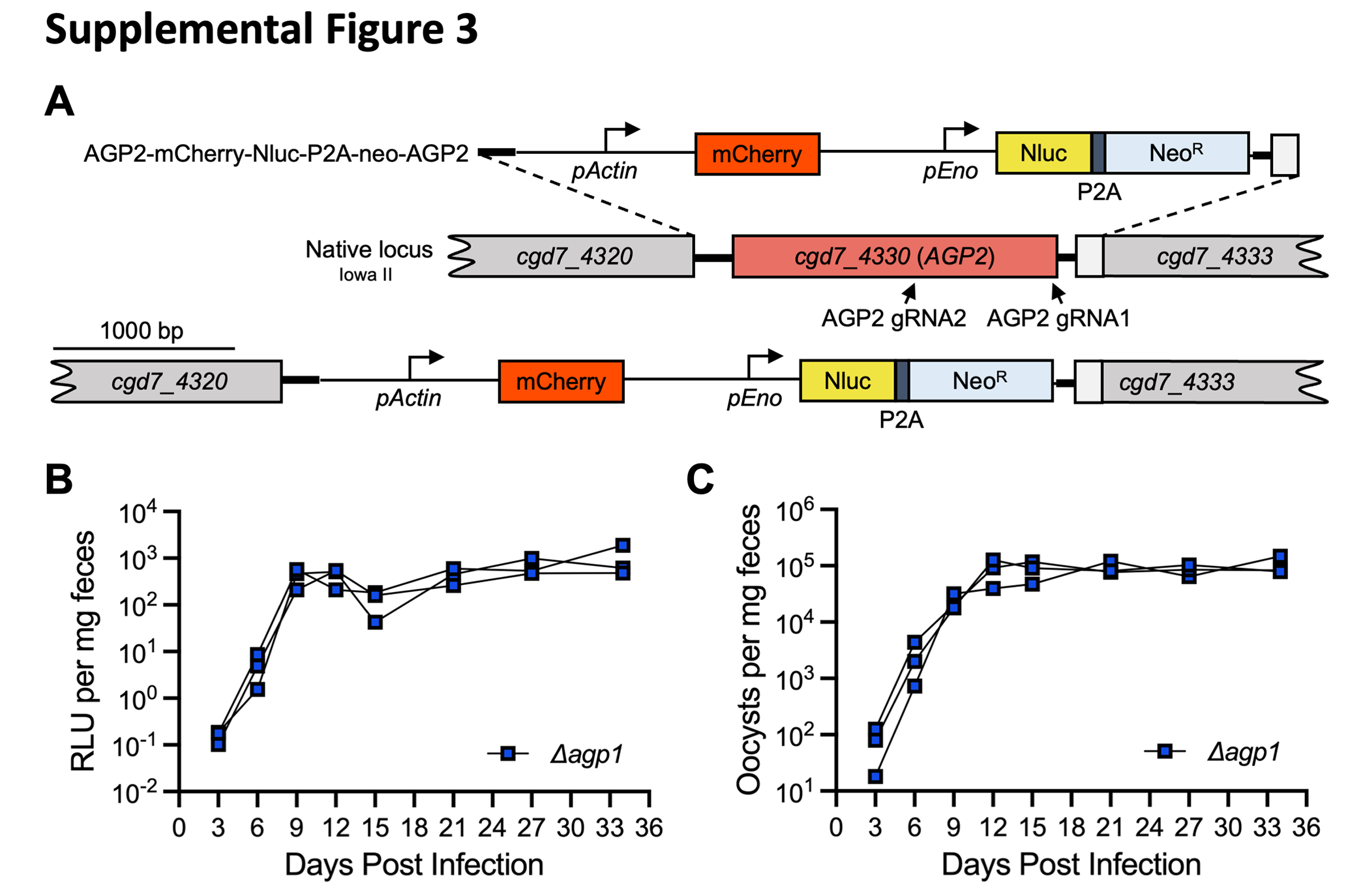

Supplement: FIG S3 [file mbio.03064-22-s0007.tif]
